# Supplementary material for: Modeling glioblastoma heterogeneity as a dynamic network of cell states
Source: Mol Syst Biol. 2021 Sep 16;17(9):e10105. doi: 10.15252/msb.202010105 (PMC8444284; doi:10.15252/msb.202010105)
Supplement: Supplementary file 5 — Source Data for Figure 3 [file MSB-17-e10105-s001.zip › Figure3A_sourcedata/GSEA_3065/nosigdb_state5.GseaPreranked.1622718435914/FELDMAN_2020_S.html]

Details for gene set FELDMAN\_2020\_S[GSEA]

|  || Dataset | state5 |
| Phenotype | NoPhenotypeAvailable |
| Upregulated in class | na\_neg |
| GeneSet | FELDMAN\_2020\_S |
| Enrichment Score (ES) | -0.49039558 |
| Normalized Enrichment Score (NES) | -1.7860163 |
| Nominal p-value | 0.0 |
| FDR q-value | 0.0024048463 |
| FWER p-Value | 0.061 |
Table: GSEA Results Summary

  

Fig 1: Enrichment plot: FELDMAN\_2020\_S      
 Profile of the Running ES Score & Positions of GeneSet Members on the Rank Ordered List

  

| PROBE | GENE SYMBOL | GENE\_TITLE | RANK IN GENE LIST | RANK METRIC SCORE | RUNNING ES | CORE ENRICHMENT || 1 | MALAT1 |  |  | 0 | 1.196 | 0.0328 | No |
| 2 | NOVA1 |  |  | 10 | 0.870 | 0.0557 | No |
| 3 | SCRG1 |  |  | 36 | 0.715 | 0.0725 | No |
| 4 | MANF |  |  | 130 | 0.499 | 0.0757 | No |
| 5 | ARL6IP1 |  |  | 232 | 0.418 | 0.0757 | No |
| 6 | ARL6IP5 |  |  | 256 | 0.406 | 0.0842 | No |
| 7 | HELLS |  |  | 280 | 0.393 | 0.0924 | No |
| 8 | CDCA7 |  |  | 495 | 0.313 | 0.0768 | No |
| 9 | BRCA1 |  |  | 522 | 0.305 | 0.0822 | No |
| 10 | ORC6 |  |  | 806 | 0.240 | 0.0568 | No |
| 11 | HES4 |  |  | 977 | 0.213 | 0.0434 | No |
| 12 | ATAD5 |  |  | 979 | 0.213 | 0.0491 | No |
| 13 | TMEM97 |  |  | 995 | 0.211 | 0.0532 | No |
| 14 | SLC20A1 |  |  | 1035 | 0.206 | 0.0545 | No |
| 15 | BAMBI |  |  | 1144 | 0.192 | 0.0475 | No |
| 16 | NASP |  |  | 1225 | 0.182 | 0.0434 | No |
| 17 | BRCA2 |  |  | 1781 | 0.128 | -0.0159 | No |
| 18 | MEST |  |  | 2059 | 0.108 | -0.0443 | No |
| 19 | HAT1 |  |  | 2459 | 0.082 | -0.0872 | No |
| 20 | AKAP12 |  |  | 2478 | 0.081 | -0.0870 | No |
| 21 | ATAD2 |  |  | 2545 | 0.077 | -0.0924 | No |
| 22 | RNASEH2A |  |  | 2560 | 0.076 | -0.0919 | No |
| 23 | ARL6IP6 |  |  | 2850 | 0.060 | -0.1229 | No |
| 24 | SRSF2 |  |  | 2908 | 0.057 | -0.1278 | No |
| 25 | SRSF7 |  |  | 3126 | 0.046 | -0.1511 | No |
| 26 | RFC4 |  |  | 3212 | 0.041 | -0.1596 | No |
| 27 | C19orf48 |  |  | 3373 | 0.033 | -0.1769 | No |
| 28 | SMC2 |  |  | 3374 | 0.032 | -0.1760 | No |
| 29 | CDC6 |  |  | 3392 | 0.032 | -0.1770 | No |
| 30 | PLAU |  |  | 3407 | 0.031 | -0.1777 | No |
| 31 | ESCO2 |  |  | 3672 | 0.021 | -0.2070 | No |
| 32 | MSH6 |  |  | 3736 | 0.019 | -0.2137 | No |
| 33 | RFC3 |  |  | 3863 | 0.013 | -0.2276 | No |
| 34 | DLGAP5 |  |  | 3907 | 0.011 | -0.2321 | No |
| 35 | CCNE2 |  |  | 4180 | -0.001 | -0.2629 | No |
| 36 | MCM4 |  |  | 4534 | -0.015 | -0.3024 | No |
| 37 | MCM10 |  |  | 4542 | -0.016 | -0.3028 | No |
| 38 | CDC45 |  |  | 4563 | -0.017 | -0.3046 | No |
| 39 | CENPK |  |  | 4675 | -0.021 | -0.3166 | No |
| 40 | CALD1 |  |  | 4714 | -0.022 | -0.3203 | No |
| 41 | CDK1 |  |  | 4766 | -0.025 | -0.3254 | No |
| 42 | FAM111B |  |  | 4792 | -0.025 | -0.3275 | No |
| 43 | MCM7 |  |  | 4986 | -0.032 | -0.3485 | No |
| 44 | GINS2 |  |  | 5024 | -0.034 | -0.3517 | No |
| 45 | HAUS8 |  |  | 5039 | -0.034 | -0.3524 | No |
| 46 | BTG1 |  |  | 5087 | -0.037 | -0.3567 | No |
| 47 | PSMC3IP |  |  | 5222 | -0.041 | -0.3707 | No |
| 48 | CCNB2 |  |  | 5382 | -0.048 | -0.3874 | No |
| 49 | POLD3 |  |  | 5393 | -0.048 | -0.3872 | No |
| 50 | SMC3 |  |  | 5536 | -0.054 | -0.4018 | No |
| 51 | CLSPN |  |  | 5614 | -0.058 | -0.4089 | No |
| 52 | MSH2 |  |  | 5621 | -0.059 | -0.4080 | No |
| 53 | CDCA3 |  |  | 5726 | -0.063 | -0.4180 | No |
| 54 | RMI2 |  |  | 5797 | -0.065 | -0.4241 | No |
| 55 | CDCA4 |  |  | 5928 | -0.071 | -0.4369 | No |
| 56 | YPEL5 |  |  | 5945 | -0.072 | -0.4368 | No |
| 57 | EXOSC9 |  |  | 6019 | -0.075 | -0.4430 | No |
| 58 | EXOSC8 |  |  | 6020 | -0.075 | -0.4409 | No |
| 59 | CHAF1A |  |  | 6157 | -0.082 | -0.4540 | No |
| 60 | PGP |  |  | 6209 | -0.086 | -0.4574 | No |
| 61 | RRM2 |  |  | 6308 | -0.091 | -0.4661 | No |
| 62 | CENPM |  |  | 6368 | -0.093 | -0.4702 | No |
| 63 | CENPA |  |  | 6374 | -0.094 | -0.4682 | No |
| 64 | ASF1B |  |  | 6400 | -0.095 | -0.4684 | No |
| 65 | ZWINT |  |  | 6453 | -0.099 | -0.4715 | No |
| 66 | DNMT1 |  |  | 6455 | -0.099 | -0.4689 | No |
| 67 | UNG |  |  | 6479 | -0.100 | -0.4688 | No |
| 68 | MCM5 |  |  | 6553 | -0.105 | -0.4742 | No |
| 69 | MAD2L1 |  |  | 6600 | -0.108 | -0.4764 | No |
| 70 | PPIF |  |  | 6616 | -0.109 | -0.4751 | No |
| 71 | LMNB1 |  |  | 6638 | -0.110 | -0.4745 | No |
| 72 | VRK1 |  |  | 6651 | -0.111 | -0.4728 | No |
| 73 | RPA2 |  |  | 6705 | -0.114 | -0.4757 | No |
| 74 | TAGLN |  |  | 6733 | -0.116 | -0.4755 | No |
| 75 | FBXO5 |  |  | 6775 | -0.119 | -0.4769 | No |
| 76 | CCDC34 |  |  | 6826 | -0.123 | -0.4792 | No |
| 77 | TGIF1 |  |  | 6926 | -0.131 | -0.4868 | Yes |
| 78 | SESN3 |  |  | 6935 | -0.132 | -0.4841 | Yes |
| 79 | DDIT4 |  |  | 6968 | -0.135 | -0.4840 | Yes |
| 80 | AURKA |  |  | 6990 | -0.136 | -0.4826 | Yes |
| 81 | CDCA7L |  |  | 7034 | -0.140 | -0.4837 | Yes |
| 82 | DNAJC9 |  |  | 7043 | -0.141 | -0.4807 | Yes |
| 83 | CCNG1 |  |  | 7061 | -0.143 | -0.4787 | Yes |
| 84 | PAIP2 |  |  | 7081 | -0.144 | -0.4769 | Yes |
| 85 | MIS18A |  |  | 7115 | -0.147 | -0.4766 | Yes |
| 86 | RPL22L1 |  |  | 7150 | -0.150 | -0.4764 | Yes |
| 87 | TFDP1 |  |  | 7187 | -0.154 | -0.4762 | Yes |
| 88 | FEN1 |  |  | 7201 | -0.155 | -0.4734 | Yes |
| 89 | GMNN |  |  | 7224 | -0.158 | -0.4716 | Yes |
| 90 | EEF1E1 |  |  | 7241 | -0.160 | -0.4690 | Yes |
| 91 | HSPB11 |  |  | 7286 | -0.166 | -0.4694 | Yes |
| 92 | EIF2S1 |  |  | 7366 | -0.177 | -0.4735 | Yes |
| 93 | MCM6 |  |  | 7375 | -0.178 | -0.4695 | Yes |
| 94 | TK1 |  |  | 7377 | -0.178 | -0.4647 | Yes |
| 95 | USP1 |  |  | 7398 | -0.181 | -0.4620 | Yes |
| 96 | MAGOHB |  |  | 7542 | -0.196 | -0.4728 | Yes |
| 97 | MCM3 |  |  | 7552 | -0.198 | -0.4684 | Yes |
| 98 | UBE2T |  |  | 7561 | -0.199 | -0.4639 | Yes |
| 99 | BEX4 |  |  | 7566 | -0.199 | -0.4589 | Yes |
| 100 | DERA |  |  | 7626 | -0.207 | -0.4599 | Yes |
| 101 | TEX30 |  |  | 7639 | -0.209 | -0.4555 | Yes |
| 102 | CARHSP1 |  |  | 7667 | -0.213 | -0.4527 | Yes |
| 103 | CENPU |  |  | 7871 | -0.248 | -0.4689 | Yes |
| 104 | DHFR |  |  | 7878 | -0.250 | -0.4627 | Yes |
| 105 | ZFAND6 |  |  | 7909 | -0.257 | -0.4590 | Yes |
| 106 | SOX4 |  |  | 7958 | -0.268 | -0.4571 | Yes |
| 107 | HMGB2 |  |  | 7973 | -0.271 | -0.4513 | Yes |
| 108 | CENPV |  |  | 8000 | -0.276 | -0.4466 | Yes |
| 109 | TPM1 |  |  | 8012 | -0.278 | -0.4402 | Yes |
| 110 | RFC2 |  |  | 8049 | -0.285 | -0.4365 | Yes |
| 111 | CTNNAL1 |  |  | 8076 | -0.292 | -0.4314 | Yes |
| 112 | PCNA |  |  | 8082 | -0.293 | -0.4239 | Yes |
| 113 | DUT |  |  | 8118 | -0.300 | -0.4197 | Yes |
| 114 | RPS27L |  |  | 8126 | -0.302 | -0.4121 | Yes |
| 115 | POLR3K |  |  | 8132 | -0.305 | -0.4043 | Yes |
| 116 | SAT1 |  |  | 8162 | -0.314 | -0.3990 | Yes |
| 117 | DCTPP1 |  |  | 8303 | -0.356 | -0.4051 | Yes |
| 118 | EIF4EBP1 |  |  | 8306 | -0.356 | -0.3955 | Yes |
| 119 | HNRNPF |  |  | 8319 | -0.360 | -0.3870 | Yes |
| 120 | PA2G4 |  |  | 8320 | -0.360 | -0.3772 | Yes |
| 121 | EIF1AX |  |  | 8331 | -0.363 | -0.3683 | Yes |
| 122 | TSC22D1 |  |  | 8334 | -0.363 | -0.3586 | Yes |
| 123 | HSPA8 |  |  | 8339 | -0.366 | -0.3490 | Yes |
| 124 | CCND1 |  |  | 8346 | -0.368 | -0.3396 | Yes |
| 125 | SLBP |  |  | 8396 | -0.387 | -0.3345 | Yes |
| 126 | LIMS1 |  |  | 8400 | -0.388 | -0.3242 | Yes |
| 127 | ACAT2 |  |  | 8440 | -0.404 | -0.3175 | Yes |
| 128 | HNRNPAB |  |  | 8441 | -0.404 | -0.3064 | Yes |
| 129 | PTTG1 |  |  | 8446 | -0.406 | -0.2957 | Yes |
| 130 | LYPD1 |  |  | 8476 | -0.422 | -0.2874 | Yes |
| 131 | CACYBP |  |  | 8486 | -0.428 | -0.2767 | Yes |
| 132 | GGCT |  |  | 8493 | -0.432 | -0.2655 | Yes |
| 133 | NCL |  |  | 8496 | -0.433 | -0.2539 | Yes |
| 134 | SNRPD1 |  |  | 8537 | -0.451 | -0.2460 | Yes |
| 135 | RAN |  |  | 8555 | -0.463 | -0.2352 | Yes |
| 136 | RPA3 |  |  | 8572 | -0.471 | -0.2241 | Yes |
| 137 | RAB13 |  |  | 8590 | -0.481 | -0.2128 | Yes |
| 138 | TYMS |  |  | 8599 | -0.488 | -0.2003 | Yes |
| 139 | SIVA1 |  |  | 8654 | -0.529 | -0.1919 | Yes |
| 140 | MARCKS |  |  | 8667 | -0.537 | -0.1785 | Yes |
| 141 | EEF2 |  |  | 8670 | -0.540 | -0.1639 | Yes |
| 142 | ANP32B |  |  | 8680 | -0.545 | -0.1500 | Yes |
| 143 | SNRPB |  |  | 8683 | -0.547 | -0.1352 | Yes |
| 144 | YWHAH |  |  | 8726 | -0.587 | -0.1238 | Yes |
| 145 | DEK |  |  | 8770 | -0.627 | -0.1115 | Yes |
| 146 | H2AFZ |  |  | 8774 | -0.630 | -0.0945 | Yes |
| 147 | PHLDA1 |  |  | 8805 | -0.655 | -0.0799 | Yes |
| 148 | RANBP1 |  |  | 8819 | -0.669 | -0.0630 | Yes |
| 149 | PTMA |  |  | 8894 | -0.764 | -0.0505 | Yes |
| 150 | EEF1A1 |  |  | 8958 | -0.915 | -0.0325 | Yes |
| 151 | FTL |  |  | 8984 | -1.286 | 0.0000 | Yes |
Table: GSEA details [plain text format]

  

Fig 2: FELDMAN\_2020\_S: Random ES distribution      
 Gene set null distribution of ES for **FELDMAN\_2020\_S**
